# Dr. Kitchen *on Nux Vomica in Dysentery*

**Published:** 1838-10-24

**Authors:** James Kitchen

**Affiliations:** Philadelphia


### Dr. Kitchen *on Nux Vomica in Dysentery.*

To the Editors of the Medical Examiner.

The more than usual prevalence of dysentery during the autumns of 1837 and '38, has elicited some remarks from a few practitioners in relation to its treatment. Among these remarks, I have been somewhat surprised to see no mention made of the *nux vomica*, which, from the success I have had with it, I have been, at times, almost inclined to look upon as a specific in this disease. It has been in use in Germany many years. The practice appears to have originated with Hagstrom, a Swedish physician, and has been highly recommended by Hufeland, Thomann, Richter, Most, Frisch, and Recamier. Vaux, of Ipswich, used it, almost specifically, in two hundred cases, (see Armstrong's Lectures;) Geddings, with success, in obstinate cases, (Am. Arch. Med. Science, Nov., 1834, and Brit. and For. Med. Rev., Jan., 1836;) Mackintosh praises it in his Practice, and refers to a trial with it, or the alcaloid, strychnine, by Graves and Stokes, published in their Clinical Reports of the Meath Hospital, Dublin. I have never used the alcaloid, the nux always answering my views; should, however, the stomach not retain the powdered nux, in the doses of five or seven grains three times a day, the alcaloid might be given, or probably the following, in preference: ℞. Nucis Vomic. ℨi. infunde in aquâ ferv. q. s. per ½ hor., ut reman.

℥vi., cola et adde Tinct. Opii ʒss. table-spoonful every two hours.

I have been induced to offer the above remarks, in addition to those already published in the Examiner and other periodicals, having been so well pleased with the remedy myself, and not having seen it referred to by any practitioner or editor; they probably have never made a trial of it, or the cases in which I administered it may have been unusually mild and well chosen for the remedy. We all have our favourite remedies and our favourite modes of practice. Meigs has his nitrous mixture, La Roche his copaiba, and Miner his capsicum: there may be cases in which all these will fail, and the nux vomica may then be worthy a trial. Without attaching undue value to the results of my own experience, the sanction of the names which I have above cited entitles the remedy in question to the notice of the profession.

James Kitchen.

*Philadelphia*, October, 1838.

